# Brain Activation During Visually Guided Finger Movements

**DOI:** 10.3389/fnhum.2020.00309

**Published:** 2020-08-14

**Authors:** Johannes Brand, Marco Piccirelli, Marie-Claude Hepp-Reymond, Kynan Eng, Lars Michels

**Affiliations:** ^1^Institute of Neuroinformatics, University of Zurich and ETH Zurich, Zurich, Switzerland; ^2^Neuroscience Center Zurich, Zurich, Switzerland; ^3^Department of Neuroradiology, University Hospital Zurich, Zurich, Switzerland; ^4^Klinisches Neurozentrum, University Hospital Zurich, Zurich, Switzerland; ^5^Centre for MR-Research, University Children’s Hospital, Zurich, Switzerland

**Keywords:** functional magnetic resonance imaging, action observation, virtual reality, visually-guided finger movements, healthy adults

## Abstract

Computer interaction *via* visually guided hand movements often employs either abstract cursor-based feedback or virtual hand (VH) representations of varying degrees of realism. The effect of changing this visual feedback in virtual reality settings is currently unknown. In this study, 19 healthy right-handed adults performed index finger movements (“action”) and observed movements (“observation”) with four different types of visual feedback: a simple circular cursor (CU), a point light (PL) pattern indicating finger joint positions, a shadow cartoon hand (SH) and a realistic VH. Finger movements were recorded using a data glove, and eye-tracking was recorded optically. We measured brain activity using functional magnetic resonance imaging (fMRI). Both action and observation conditions showed stronger fMRI signal responses in the occipitotemporal cortex compared to baseline. The action conditions additionally elicited elevated bilateral activations in motor, somatosensory, parietal, and cerebellar regions. For both conditions, feedback of a hand with a moving finger (SH, VH) led to higher activations than CU or PL feedback, specifically in early visual regions and the occipitotemporal cortex. Our results show the stronger recruitment of a network of cortical regions during visually guided finger movements with human hand feedback when compared to a visually incomplete hand and abstract feedback. This information could have implications for the design of visually guided tasks involving human body parts in both research and application or training-related paradigms.

## Introduction

Visually guided arm and hand movements are common in many computer-mediated interactions for motor research, training, and entertainment (Mattar and Gribble, 2005; Archambault et al., 2015). Such interactions typically use cursors (Oreja-Guevara et al., 2004; Veilleux and Proteau, 2011), often in the form of simple circles, to provide feedback on the current position of the endpoint of the hand and arm. Feedback in the form of a cursor carries enough information for the central nervous system to plan and execute the required movements if the task accomplishment only depends on the position of the cursor. However, visual cues that are more realistic could conceivably improve performance in these movement scenarios. Previous studies showed that seeing a hand for a short period at movement initiation, either real (Veilleux and Proteau, 2011) or virtual (Sober and Sabes, 2005), led to higher accuracy during goal-directed target reaching. Based on these results, we previously experimented on adults comparing different forms of online feedback during visually guided finger movements in a finger-reaching task. We found that providing hand, virtual hand (VH) or shadow hand (SH) instead of point light (PL) or cursor (CU) feedback resulted in significantly faster movement initiation for three out of the four comparisons (not for VH vs. PL; Brand et al., 2016). Hence, the results of our previous study suggested that hand feedback might play an important role in visually guided movement control and in its underlying brain processes. However, no study has investigated the differences in brain activation between hand and cursor feedback during reaching movements.

Also, it has not yet been determined whether observing a hand movement will lead to the same or different brain activations than performing the same hand movement. Rizzolatti and collaborators found in monkey ventral premotor (PMv or F5) and in the inferior parietal lobule (IPL) so-called “mirror neurons,” which fired not only during the monkey’s hand movement but also during observation of the same movement performed by the experimenter (Rizzolatti et al., 1996, 2006). The question of whether these cortical two brain regions in humans would also respond to observation of the hand movements has been investigated in a wealth of brain imaging publications. One of the first studies with functional magnetic resonance imaging (fMRI) demonstrated that observation leads to activation in several regions, namely bilateral premotor and also parietal cortices (Buccino et al., 2001). Iacoboni et al. (1999) also revealed that two human brain regions (i.e., inferior frontal and inferior parietal cortex) had mirror neurons properties, as they responded similarly for movement observation, imitation, and execution. These regions were named the mirror neuron system (MNS; Rizzolatti et al., 2001) or the action observation network (Buccino et al., 2001). Several further publications also reported activations only for observation of hand actions outside of the MNS, such as in the sensorimotor cortex (Szameitat et al., 2012) as well as in visual regions (Orlov et al., 2014; Bracci et al., 2015).

In an early positron emission tomography experiment (PET), observation of grasping movements was delivered for three different types of hand feedback: real hand (RH), high-quality visual reality VRH, and low-quality VRL feedback (Perani et al., 2001). For all three experimental conditions, a large number of brain regions, some visual and others in the parietal lobe were activated. Enhancement in brain activation only occurred in the right IPL for watching real hand compared to virtual hand actions. Yet, an agent performed the movements in the scanner room and this could have induced other effects than purely feedback related ones. Other more recent publications also reported activation not only in the IPL but also in the anterior intraparietal sulcus (IPS) and in the prefrontal cortex in visually guided grasping and in hand movements (Shmuelof and Zohary, 2006; Pilgramm et al., 2009).

There is further evidence that action observation of moving objects and hands lead to neural responses outside of the MNS (Engel et al., 2008) in higher visual cortex (Kaneko et al., 2015) and sensorimotor areas (Szameitat et al., 2012). Using fMRI, it was found that the lateral occipital cortex (LOC) is involved in the observation of stimuli including hands (Grill-Spector et al., 2001; Culham et al., 2003). It was also reported that the lateral occipitotemporal cortex (LOTC) shows closely overlapping fMRI signals for observation of hands and tools (Bracci et al., 2012). Further, the visual area hMT+ plays a role in biological motion perception (Grezes, 1998; Grossman and Blake, 2002; Michels et al., 2005), including visually guided hand movements (Oreja-Guevara et al., 2004). The extrastriate body area (EBA)—a part of the occipitotemporal cortex (OTC)—is known for responding selectively to the observation of human bodies or body parts, including hands (Downing et al., 2001; Taylor et al., 2007). Importantly, the EBA can distinguish between self-generated and other generated hand movements, indicating that this region is not only responsive during the observation of visual stimuli (David et al., 2007).

In the present fMRI study, we investigated brain activations associated with various visual feedback conditions actively and observed performed visually guided finger movements. By applying a virtual reality mediated feedback during the action, we could test for the impact of self-performed hand movements using very sparse (cursor and point-light) to realistic displays of index finger movements (shadow and virtual hand). We expected that execution as well as observation of hand movements would lead to the strongest activation during the realistic display of a hand.

## Materials and Methods

### Participants

Twenty-seven healthy paid volunteers participated in the study. We excluded eight subjects for the following reasons: two volunteers were co-authors, that were aware of the study hypotheses; two other volunteers demonstrated significant head motion (>3 mm in translation); in another four volunteers, coil artifacts were observed in the structural MRI. The remaining 19 subjects (seven females) were all right-handed as evaluated using the Edinburgh inventory (Oldfield, 1971) and had a normal or corrected-to-normal vision. Subjects were on average 27.9 years (SD 7.4 years) old and provided written informed consent before participation. Our study was accepted by the Kantonale Ethikkommission Zürich and experiments were conducted in compliance with the Declaration of Helsinki.

### Technical Setup

**Figure 1:**
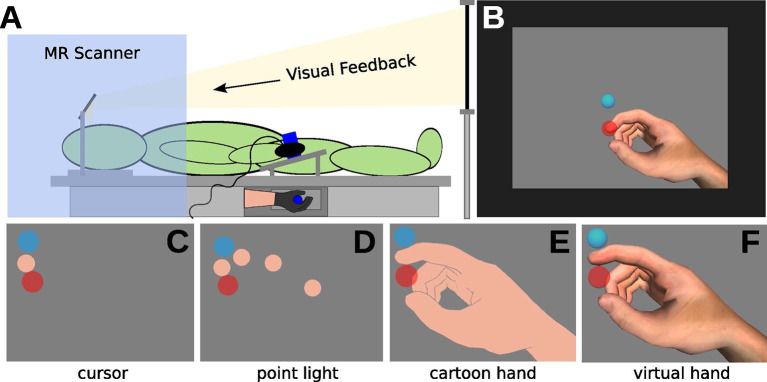
Illustration of the experimental setup. **(A)** In the MRI, participants wearing a data glove and grasping a tube (with the right hand). Visual feedback was delivered *via* mirror projection from a monitor. **(B)** Visual feedback consisted of the display of the starting position (light-blue circle), of the movement cursor (skin-colored circle, approximately Type III on the Fitzpatrick scale), and the target (red circle) on grey background. **(C–F)** The experiment comprised eight conditions, with four different types of visual feedback. **(C)** Cursor (CU), **(D)** point light (PL), **(E)** shadow hand (SH), and **(F)** virtual hand (VH) feedback.

The experiment setup is visualized in Figure 1. Parts of the setup have previously been described in our previous study (Brand et al., 2016). The experiment was performed with subjects lying in a wide bore (70 cm diameter) magnetic resonance scanner. Subjects fixated a rigid plastic tube in a power grip with their right hand. The tube was fixed to the scanner bed to assist in keeping the hand in a comfortable neutral position. This ensured that hand and finger positions were approximately consistent across participants. Tubes with three different diameters (5.1, 4.7, and 4.3 cm) were used to adjust for varying hand sizes. Index finger movements were recorded using a 5DT Data Glove 5 MRI (5DT Inc., Irvine, CA, USA)^1^. We used Unity3D (version 3, Unity Technologies, San Francisco, CA, USA)^2^ for data acquisition, data processing, and presentation of real-time visual feedback. Movement as well as task feedback were presented in real-time on an LCD monitor, which participants were able to observe *via* a mirror. An Eyelink 1000 long-range video oculography system (SR-Research Limited, ON, Canada)^3^ was mounted underneath the monitor to record the movements of one eye. A nine-point calibration routine for the tracker was executed at the beginning of the experiment; data were recorded at 500 Hz.

Data from the sensor glove was acquired at 75 Hz. A moving average filter was applied for smoothing the input data over a 100 ms window. Smoothing was employed to remove the high-frequency noise from the sensory input, but we also added a delay of 50 ms to the recorded user input, which was not noticed by participants, as stated after the experiment. At every smoothed input sample, the three index finger joint angles were inferred using a lookup table. The angles were then applied to a realistic virtual hand model. Realistic joint angles of the finger flexion-extension movement task were acquired using motion capturing (Vicon, USA) in a separate single participant study preceding this experiment. In the pre-study, the single participant performed the movement task outside of the scanner and the joint angle mapping data for the movement was stored in the lookup table. Before the experiment, the sensor glove was calibrated for every subject at 100% and 0% index finger extension. The task was performed in the 95% to 5% index finger extension range. We report distances relative to this movement range in the course of this article.

A realistic virtual hand model from the 5DT data glove software was used (2,450 polygons) for visual feedback of the finger movements. Virtual spheres, sized to approximate the thickness of the real index finger, were placed on the virtual index fingertip and finger joint positions. Four distinct hand feedback types were defined:

CU (Figure 1C): invisible hand model, the tip of the index finger visible as a sphere (flat flesh-colored shading, orthographic 2D projection)

PL (Figure 1D): invisible hand model, spheres on the joints of the index finger visible (flat flesh-colored shading, orthographic 2D projection)

SH (Figure 1E): visible hand model with moving index finger, all spheres invisible (flat flesh-colored shading, perspective 3D projection)

VH (Figure 1F): visible RH model with moving index finger, all spheres invisible (realistic shading, perspective 3D projection)

We adjusted the visual angle of the task range on the screen for every subject to match the visual angle of the real finger movements in the lying task position.

### Experimental Protocol

Subjects were instructed to either observe or control the movements of the virtual index finger (hand or circles) projected on the screen. During the action conditions, participants controlled the virtual hand and circle stimuli by extending and flexing their right index finger. They were instructed to move the index finger and to stabilize the other fingers by holding the tube. During the observation conditions, subjects were resting their hand by holding the tube and they watched the pre-recorded and animated movements of the virtual effector on the screen. To control for finger and eye movements, we recorded sensor glove and eye-tracker data throughout the whole experiment (during the action as well as observation conditions). The task started with moving the cursor represented by a blue circle into the starting position (Figure 1B). After two s, the trial started with a red target circle appearing at a pseudo-random location (between 40–50% finger extensions). Starting and target position circles were displayed in different colors to facilitate distinguishing between the two visual positions, especially in observation conditions. In action conditions, participants were instructed to move the cursor as fast and accurately as possible to the target, and immediately back to the starting position. Each trial lasted 2 s, with the target circle disappearing after 1 s. The trials were grouped in blocks of nine and each block lasted 22 s comprised of 2 s task instruction, 2 s for moving the cursor into the starting position, and followed by nine trials of 2 s duration each. The blocks were interleaved with in-between resting periods of pseudo-random length (7–9 s, average 8 s) showing a blue fixation cross. The whole experiment consisted of eight conditions, each comprising ten blocks. Each condition contained a different feedback type for the task (Figures 1C–F) and additionally differed in whether subjects observed or performed (action) the movements.

Randomly, one action and one observation condition were assigned to one of four scanning runs, such that by pseudo-random selection either the same feedback type or two different feedback types were used within one run. Within the runs, respective action and the observation condition blocks were presented in random order. To instruct subjects on the task, either the word “action” in red or the word “observation” in green was presented for 2 s before each block. In between runs, participants were allowed to take a short break, if required. The whole experiment lasted for approximately 1 h, including setup time.

### Behavioral and Eye Data Analysis

For behavioral and eye movement data analysis we used the same methodology as in our previously published behavioral study (Brand et al., 2016). Please refer to the previously published article for a detailed description of the procedure. In total, for behavioral data, we calculated four psychometric parameters for each index finger movement: movement amplitude, movement extent error (Euclidean distance between the virtual index fingertip and the target center), total movement time, and reaction time. Reaction time was operationalized as the time between stimulus presentation and visual movement onset. Movement onset was detected when 10% of the distance from the starting position to the target distance was exceeded. The parameter of movement onset and therefore reaction time includes the 50 ms lag of the moving average filter. For eye movements, we calculated seven parameters: number of saccades, fixations, blinks, horizontal and vertical gaze amplitude, median horizontal, and vertical gaze velocity. Statistical testing for the factor Condition was performed using the appropriate parametric (one-way repeated measures ANOVA or paired *t*-test) or non-parametric test (Friedman test or Wilcoxon signed-rank test).

### fMRI Data Acquisition

Scans were acquired using a Philips Ingenia 3.0 Tesla MR scanner (Philips Healthcare, Best, The Netherlands) with a Philips 32-element head coil. Functional blood oxygenation level-dependent (BOLD) sensitive images were obtained using a single-shot gradient-echo EPI (Mansfield, 1977) pulse sequence (slices = 32, slice scan order: interleaved, repetition time = 2.2 s, echo time = 35 ms, flip angle = 90°, field of view = 230 × 230 mm^2^, reconstruction voxel size = 2.9 × 2.9 × 4.4 mm^3^, scan matrix 64 × 64). The images were recorded in an oblique axial orientation to reduce signal dropout and covered the whole brain. To homogenize the BOLD sensitivity of the fMRI scan, we applied sensitivity encoding with a reduction factor of one. Additionally, the possible number of slices acquired within one TR was maximized. Following the functional scans an anatomical scan was acquired, using a sagittal 3D T1-weighted gradient-echo sequence (slices = 170, repetition time = 8.2 ms, echo time = 3.8 ms, flip angle = 8 degrees, field of view = 240 mm, voxel size = 0.94 × 0.94 × 1 mm, scan matrix 240 × 240). All images covered the whole brain. Each run lasted 10 min and 49 s and contained an observation and action condition.

### fMRI Data Analysis

We analysed the fMRI data with SPM8^4^. We discarded the first three dummy images of every fMRI run to obtain a magnetization steady-state and performed the following pre-processing steps: realignment to the calculated mean image, segmentation, normalization to the Montreal Neurological Institute (MNI) template and smoothing by an isotropic Gaussian kernel of 6 mm full-width at half maximum. The data was high-pass filtered with a cut-off frequency of 1/128 Hz. Subsequently, we performed for each participant a first-level trial-based analysis with a general linear model (GLM) as implemented in SPM8. For each subject, the design matrix consisted of eight regressors, corresponding to the trial onsets and durations (2 s) of the eight conditions, four amplitude parametric modulators, coding movement amplitude in the action conditions as described in the behavioral data analysis section and four constants for each fMRI run. The amplitude parametric modulators were included to remove global amplitude effects from the signal. The trial-based assessment of movement amplitude has been described previously (Brand et al., 2016). Subsequently, the regressors and parametric modulators were convolved with the hemodynamic response function. Also, we used eye movement parameters (i.e., number and durations of saccades, fixations, eye blinks as well as the amplitude and velocity in the translational and horizontal direction) as nuisance variables (covariate of no interest) in the second-level analysis.

The fit of the GLM to the observed activity yielded parameter estimates for every subject, voxel, and regressor or parametric modulator. The beta values represent an estimate of activation for regressors and an interaction estimate of regressor activation with parameter value for parametric modulators. We then calculated the t-contrasts of each estimated regressor or parametric modulator compared to baseline. All resting periods of 7–9 s (average 8 s) in between blocks and 30 s at the beginning and the end of each fMRI run served as baseline for the GLM.

We entered the resulting beta images into a second-level random-effects group analysis. The second-level design matrix consisted of eight regressors stemming from two factors of two and four levels, respectively. In particular, we used a 2 × 4 factorial design for the two-way ANCOVA with the factors *Condition* (action and observation) and *Feedback* (VH, SH, PL, and CU) to examine main and interaction effects. We also performed a conjunction analysis for factor *Condition*. As described, eye movement parameters were used as a nuisance variable. In the case of significant main effects (*F*-test), *post hoc* t-contrasts (paired, one-tailed) were performed. General task activations were assessed by calculating t-contrasts of combined action or observation regressors to baseline. For all analyses, we used the SPM implemented family-wise error (FWE) correction to find activated voxel-clusters with an overall type I error level of *p* < 0.05. The results were visualized on a cortical surface using the (PALS)-B12 atlas (Van Essen, 2005) and the software Caret (Van Essen et al., 2001).

We defined four literature-driven regions of interest (ROIs) in the left hemisphere with center coordinates taken from previous publications (see Table 1). MNI (MNI space) transformed coordinates for the EBA were taken from Orlov et al. (2010), for area hMT+ from Spiridon et al. (2006), for the LOC from Grill-Spector et al. (2000) and the LOTC from Bracci et al. (2010). To compare activations in the ROIs across conditions first-level beta values were extracted using MarsBaR 0.43^5^ from 6 mm spheres around the peak coordinates for every subject and regressor. The ROI data were compared for feedback-specific effects by one-way repeated-measures ANOVAs and paired *t*-tests or by their non-parametric counterparts Friedman-tests and paired Wilcoxon signed-rank tests.

**Table 1:** MNI coordinates used as centers for the ROI analysis with corresponding references.

	Montreal Neurological Institute space			
Brain region	*x*	*y*	*z*	Source
EBA (part of OTC)	−48.3	−77.1	7.2	Orlov et al. (2010)
hMT+	−54.0	−74.3	13.8	Spiridon et al. (2006)
LOC	−46.3	−70.3	−0.2	Grill-Spector et al. (2000)
LOTC	−49.5	−70.1	2.1	Bracci et al. (2010)

*EBA, extrastriate body area as part of the; OTC, occipitotemporal complex; hMT+, human area MT+; LOC, lateral occipital complex; LOTC, lateral occipitotemporal cortex*.

## Results

### Behavioral Data

Overall, participants performed similar movements for the four visual feedback action conditions. In our previous article on 26 participants, we found faster reaction times (for three out of four comparisons), larger movement amplitudes, and larger movement extent errors for the hand compared to the circle conditions (Brand et al., 2016). Likewise, the behavioural results of the 19 participants studied in this article, revealed significant differences in one-way repeated measures ANOVAs for factor *Condition* in movement amplitude (*F*_(3,75)_ = 119.66, *p* < 0.001), movement extent error (*F*_(3,75)_ = 129.03, *p* < 0.001), and reaction time (*F*_(3,75)_ = 7.24, *p* < 0.001), but no significant effect on movement time.

Additionally, in line with our previous report, movement amplitude was significantly larger in the two hand feedback conditions than in the two circle conditions (VH > PL, *t* = 12.40, *p* < 0.001; VH > CU, *t* = 13.18, *p* < 0.001; SH > PL, *t* = 11.77, *p* < 0.001; all *t*-tests; SH > CU, *V* = 190.00, *p* < 0.001; Wilcoxon signed-rank test). Accordingly was movement extent error (VH > PL, *t* = 12.89, *p* < 0.001; VH > CU, *t* = 13.32, *p* < 0.001; SH > PL, *t* = 11.98, *p* < 0.001; SH > CU, *t* = 14.11, *p* < 0.001; all *t*-test).

As in our previous article, reaction time was significantly shorter for SH than for CU and point light conditions (PL > SH, *t* = 3.00, *p* = 0.010; CU > SH, *t* = 4.34, *p* = 0.002; *t*-tests). However, different to our previous study with 26 participants, in this study we could not find a significant effect on reaction time for virtual hand compared to cursor (CU > VH, *t* = 2.96, *p* = 0.050; *t*-test). All tests were Bonferroni corrected for multiple comparisons.

To support our neuroimaging hypothesis, which compared both hand conditions combined to both circle conditions combined, we also investigated this contrast in behavior. In Bonferroni corrected *t*-tests, we found significantly larger movement amplitude (hands > circles, *t* = 16.02, *p* < 0.001) and movement extent error (hands > circles, *t* = 16.03, *p* < 0.001) as well as shorter reaction time for hand compared to circle conditions (circles > hands, *t* = 4.07, *p* < 0.001).

### Eye-Tracking Data

The median position of the subjects’ tracked eye stayed close to the endpoint of the controlled effector (circle or index finger) for all conditions. In median trajectories, differences between conditions and overruns were small, while differences between individuals were large. We investigated the differences between conditions with parameters acquired from the eye-tracking trace trial-by-trial. As the data was not normally distributed, non-parametric tests (Friedman or Wilcoxon signed-rank tests) were used and the *p*-values were Bonferroni corrected for the number of tests performed. We did not find any differences for factor *Feedback* for none of the assessed parameters, neither for action nor for observation. However, for factor *Condition* (separating action from observation conditions), the number of saccades, fixations, and blinks were significantly different (all *p* < 0.05, Friedman tests). *Post hoc* analyses with Wilcoxon signed-rank tests revealed a greater number of saccades (*V* = 175, *p* < 0.001), fixations (*V* = 179, *p* < 0.001) and blinks (*V* = 190, *p* < 0.001) during observation than action.

### fMRI Data

#### General Task Activation—Whole-Brain Analysis

**Figure 2:**
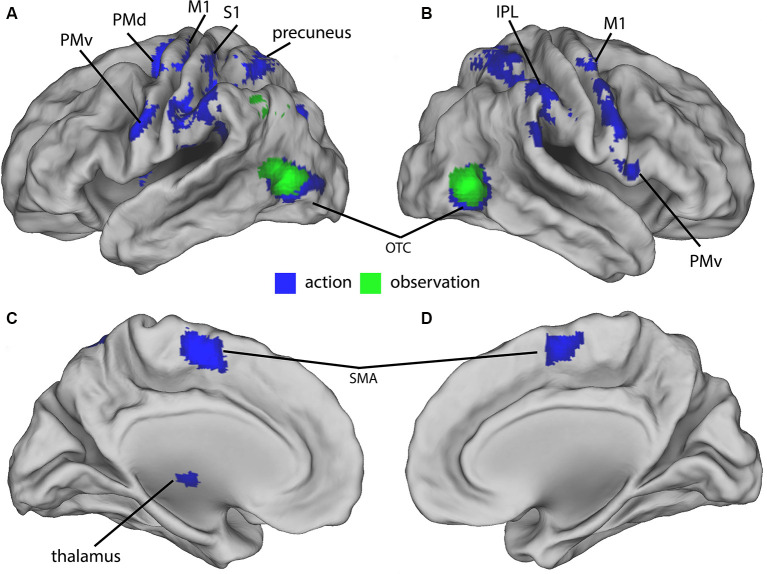
Group-activations of action > baseline (blue) and of observation, > baseline (green) both overlaid on a rendered brain. **(A)** Left lateral, **(B)** right lateral, **(C)** left medial, and **(D)** right medial view. Primary motor (M1) and somatosensory (S1) cortex, dorsal (PMd) and ventral (PMv) premotor cortex, supplementary motor area (SMA), precuneus, inferior parietal lobule (IPL), occipitotemporal cortex (OTC), and cerebellum predominantly activated. Activations are shown at *p* < 0.05 (FWE corrected).

In the final sample of subjects (*n* = 19), head movements were small (all slice-specific translation values < 2 mm; rotation <2 degrees). The 2 × 4 ANCOVA yielded significant main effects for the factors *Condition* (*F*_(1,134)_ = 11.3, *p* < 0.001, *F*-test) and *Feedback* (*F*_(3,134)_ = 5.7, *p* < 0.001, *F*-test) but no interaction effects. We first analyzed the BOLD signal of all action and observation conditions and compared them to the baseline to assess general task effects. Both action and observation conditions activated the bilateral OTC (Figure 2). Also, the conjunction analysis revealed a similar bilateral cluster in the OTC shown in (Figure 2). The action conditions additionally activated bilateral cortical (motor, premotor, somatosensory, and parietal) and cerebellar regions (not shown) as well as ipsilateral thalamic regions (Figure 2). These were located in the primary motor and somatosensory cortex, dorsal premotor cortex (PMd), PMv, supplementary motor area (SMA), precuneus, IPL, and in the cerebellum.

#### Feedback Effect—Whole-Brain Analysis

**Figure 3:**
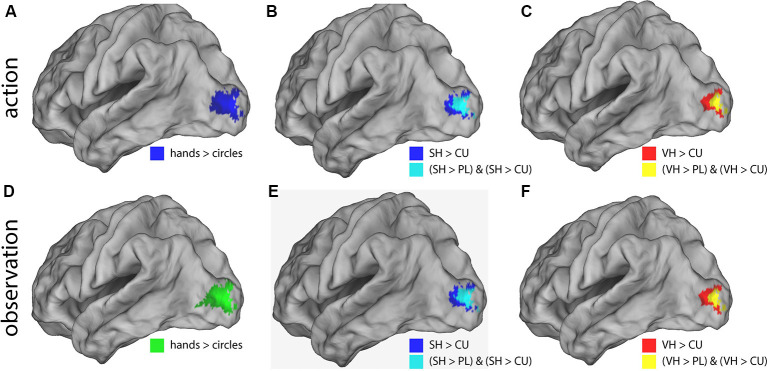
Illustration of the significant BOLD signal differences comparing feedback types. Activations are shown for both action **(A–C)** and observation **(D–F)** conditions overlaid on a left lateral rendered brain. Right-hemispheric activations were small and not shown. Activations of all plots were FWE corrected (*p* < 0.05). This figure illustrates that during the action and observation realistic displays of a human hand (SH and VH) activate the visual cortex more strongly than point-light (PL) or cursor (CU) displays.

*Post hoc* t-contrasts (paired, one-tailed) were then performed for the factor *Feedback* the following *post hoc* T-contrasts for each condition: VH vs. SH, SH vs. PL and PL vs. CU. Significant activations were only found for SH > PL and PL > CU in both action and observation conditions (Figure 3 and Table 2).

**Table 2:** Peak activations for the action and observation conditions.

Action						
		Cluster	Peak	MNI		
Brain region	Hem.	size	*t*-value	*X*	*Y*	*Z*
Hands > circles						
V3d, hOc3d	left	601	9.6	−20	−96	6
VH − CU and VH − (CU + PL)						
V3d, hOc3d (middle occipital)	left	383	7.6	−20	−96	6
V3v, hOc3v (calcarine)	left		6.1	−14	−94	−10
SH − CU and SH − (CU + PL)						
V3d, hOc3d (middle occipital)	left	445	7.8	−20	−96	6
hOc4lp (middle occipital)	left		6.8	−28	−88	6
V3v, hOc3v (calcarine)	left		5.3	−12	−92	−8
Observation						
Hands > circles						
V3d, hOc3d (middle occipital)	left	584	12.1	−20	−96	6
hOc4la (middle occipital)	left		4.7	−44	−82	−2
VH − CU and VH − (CU + PL)						
V3d, hOc3d (middle occipital)	left	375	8.9	−20	−96	6
SH − CU and SH − (CU + PL)						
V3d, hOc3d (middle occipital)	left	606	10.9	−20	−96	6
V2, hOc2 (calcarine)	left		6.3	−8	−94	−10
hOc4la (middle occipital)	left		4.8	−44	−82	0

*Hands summarize VH and SH. Circles summarize PL and CU. All results presented at *p* < 0.05 (FWE corrected) and peak voxels reported in MNI coordinates. The reported brain regions correspond to regions of the Anatomy Toolbox 2.2 (Eickhoff et al., 2005)*.

The SH > PL comparison significantly activated the lingual gyrus in both action and observation conditions. In action, the single activation cluster was larger in the number of activated voxels compared to observation and extended into the inferior occipital gyrus.

PL > CU activated the lingual and fusiform gyrus for action and observation with the peak for action lying in the fusiform gyrus and the one for observation in the lingual gyrus (Figure 3 and Table 2).

SH > PL and PL > CU had no overlap in both action and observation conditions.

To investigate the effect of seeing a hand on activations, we compared hands (VH, SH) vs. circles (PL, CU) by combining both hands as well as both circle conditions. The contrast hands > circles yielded significant activations in several brain regions (Figure 3 and Table 2).

The hands > circles contrasts of both action and observation had an activation cluster in the left (contralateral) occipital lobe, mainly in the lingual gyrus. For the action conditions, this cluster extended into the fusiform gyrus, middle temporal gyrus, and cuneus. Additional activation clusters for action were found in the left precuneus and cuneus. The hands > circles activations contained all SH > PL activations, but not all PL > CU activations.

In summary, we found stronger activations for feedback of hands than circles during both action and observation conditions, with larger activations in action. Many of these regions were also significantly activated by the SH > PL comparison. Disjoint from the SH > PL activations we found significantly higher BOLD signals for PL > CU feedback in early visual regions.

### ROI Analysis

**Figure 4:**
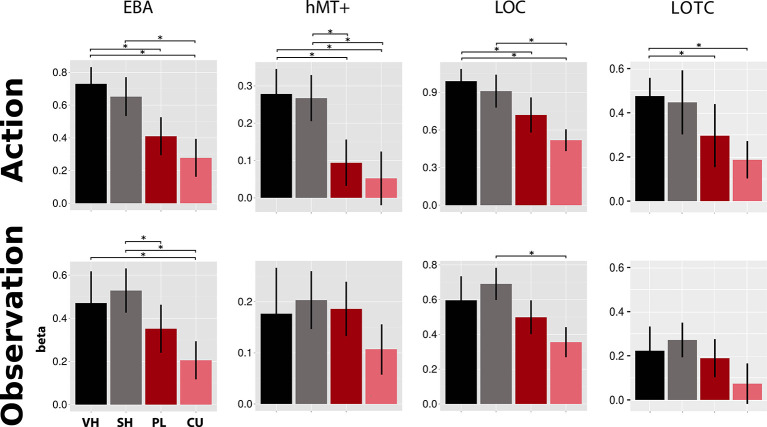
Activations in four literature-driven (EBA, hMT+, LOC, and LOTC, see Table 1) left-hemispheric ROIs. Beta value means and standard errors extracted for VH (black), SH (gray), PL (dark red), and CU (light red) conditions during both action (top) and observation (bottom). Asterisks indicate significant changes between conditions (*p* < 0.05). Abbreviations: EBA, extrastriate body area; hMT+, human area MT+; LOC, lateral occipital complex; LOTC, lateral occipitotemporal complex. This figure indicates that during the action and observation realistic displays of a human hand (SH and VH) activate the literature-driven ROIs more strongly than point-light (PL) or cursor (CU) displays.

As shown in Figure 4, the EBA showed significant activation differences between hand and cursor conditions in action (VH vs. CU, *t* = 4.49, *p* = 0.002; SH vs. CU, *t* = 3.84, *p* = 0.007; *t*-tests) and observation (VH vs. CU, *V* = 164, *p* = 0.024; Wilcoxon signed-rank test; SH vs. CU, *t* = 6.24, *p* < 0.001; *t*-test). We also found a significant difference between VH and PL in action (*V* = 183, *p* < 0.001; Wilcoxon signed-rank test) and between SH and PL in observation (*t* = 3.28, *p* = 0.025; *t*-test).

Activation in hMT+ was higher for hands than for circles in action (VH vs. PL, *V* = 180, *p* < 0.001; VH vs. CU, *V* = 162, *p* = 0.032; Wilcoxon signed-rank tests; SH vs. PL, *t* = 3.00, *p* = 0.046; *t*-test; SH vs. CU, *V* = 173, *p* = 0.005; Wilcoxon signed-rank test).The LOC showed a decreasing activation gradient from VH to CU conditions in action (Figure 4). Significant differences were found between the two hand conditions and the CU (VH vs. CU, *t* = 4.97, *p* < 0.001; *t*-test; SH vs. CU, *V* = 176, *p* = 0.003; Wilcoxon signed-rank test) and for VH compared to PL (*V* = 163, *p* = 0.027; Wilcoxon signed-rank test). In observation, differences were only significant for SH vs. CU (*t* = 4.92, *p* < 0.001; *t*-test).

For the LOTC, we found stronger fMRI responses for the *Feedback* types “hands” compared to “circles” for both action and observation. Significant differences were seen for action for two comparisons (VH vs. PL, *V* = 164, *p* < 0.024; Wilcoxon signed-rank test; VH vs. CU, *t* = 3.74 *p* = 0.009; *t*-test).

## Discussion

The main aim of this study was to examine whether neuronal engagement depends on the realism of a human hand during action and observation. We found main effects of *Feedback* and *Condition*, but no interaction effects. This indicates that fMRI signal responses were different in action (visually guided hand movements) and observation and differed between feedback types. However, BOLD responses were similarly modulated by the different feedback types for action and observation. As expected, seeing a hand representation while performing index finger movements activated the known relevant sensorimotor and visual brain regions (when controlling for movement amplitude and eye movement parameters). Activations in visual regions were stronger for VH movements than for moving PL. The difference between the two types of hand feedback representations did not lead to measurable differences in brain activation. The observation did not activate as many regions as action and the activation was weaker during displays of moving PL. These results suggest that the engagement of the brain during virtually guided index finger movements is stronger and spatially more extended during the display of a hand compared to various PL displays. Unexpectedly, regions of the MNS were not seen during observation using our experimental design. Our results thus clearly suggest that visual feedback of virtually guided hand movements can robustly activate the MNS and the visual cortex. This finding might help to design experiments in which robust neuronal responses of the visual cortex are required.

### Action vs. Observation: Regional Differences in Brain Activation

In all four literature-derived ROIs (EBA, hMT+, LOC, and LOTC), we found stronger activation for a moving hand (VH and SH) compared to moving circle stimuli (PL and CU). For action and observation, the whole-brain comparison between hand (VH or SH) and circle (PL or CU) activated left occipital and occipitotemporal (LOC and LOTC) regions. The OTC is known for processing visual information about the human body (Downing et al., 2001, 2006; Taylor et al., 2007; Lingnau and Downing, 2015). This region has also been associated with action observation and performance (Caspers et al., 2010; Lingnau and Downing, 2015) and has been proposed as an MNS region (Molenberghs et al., 2012a,b). Regions for visual processing of the body or body parts are known to exist in EBA (Downing et al., 2001) and FBA (Peelen and Downing, 2005), which are both parts of the OTC. In our experiment, a hand display activates EBA more strongly than the two types of circle conditions during both action and observation. This is consistent with studies reporting EBA activation for observation of static as well as moving hands (Taylor et al., 2007; Op de Beeck et al., 2010; Orlov et al., 2010). Nevertheless, these studies only compared observation of hands to observation of objects. Our results extend their findings, as in our study EBA was active during both action and observation of hands compared to PL displays.

We found that only during the action, hMT+ was activated for all hands and circle conditions. Studies have revealed that sub-regions of hMT+ also respond to the motion in other sensory modalities, such as tactile motion (Lingnau and Downing, 2015). Thus, being considered a motion-processing region, one would expect hMT+ to be activated equally for all feedback types, and both action and observation. However, activations for PL and CU were significantly smaller during action compared to those for hand conditions. This finding could be related to a previous study that found hMT+ responding more strongly to the motion of body parts than to other objects (Spiridon et al., 2006). There is evidence that macaque’s MT is involved when motion energy is integrated into the visual field (global motion; Salzman et al., 1992; Pasternak and Merigan, 1994), which is especially the case for the hand conditions in our experiment. Further, it has been proposed that hMT+ activations depend on the level of implicit motion (Kable et al., 2002). Hence, processing of motion from own actions—like in our action condition—might be different from the processing of motion from pre-recorded actions like in our observation, as the amount of implicit motion is absent in this condition. Feedback from own performed motion is required for visuomotor control by enhancing internal movement models (Shadmehr and Krakauer, 2008).

Especially for action, LOC activations were significantly stronger for the hand than circle conditions. Our results extend previous imaging findings showing that the LOC responds more to real objects compared to scrambled objects (Grill-Spector et al., 2001). During the observation, activation was the strongest for SH feedback and significantly different from CU feedback. Thus, any difference could be explained by the presence of a moving hand.

In line with previous reports (Bracci et al., 2012, 2018), we found hand-selective responses in the left LOTC, most pronounced for the VH condition during both *Feedback* types. Our results thus indicate that brain activation is driven by the observation of whole integrated real finger movements and visually guided real finger movements, and not by the movements of subparts of the hand (abstract subparts of the finger).

### Behavioral and Eye-Tracking Data

We found differences between the four action conditions in three psychophysical parameters: finger movement amplitude, extent error, and reaction time. Some of these results were previously presented (Brand et al., 2016). To remove the influence of behavioral effects on the fMRI data, we added a movement amplitude regressor of no interest as a parametric modulator to the first-level fMRI model. Our eye-tracking data analyses did not yield any significant differences between the four feedback conditions. Thus, any behavioral differences in amplitude and reaction time cannot be explained by differences in eye movements. However, differences between action and observation conditions were found in the number of saccades, fixations, and blinks, being larger for observation. This may be due to more focused attention during action than during observation. Eye movements are known to modulate activity in the frontal brain areas, such as the frontal eye field, as well as in parietal brain regions. The frontal eye field contains visual, motor, and visuomotor cells (Bruce and Goldberg, 1985) essential for the preparation and triggering of eye movements. Transcranial direct current stimulation during saccade preparation over the IPS can alter general performance, e.g., during a discrimination task, which is not necessarily the case for the frontal eye field (Neggers et al., 2007; Van Ettinger-Veenstra et al., 2009). As we observed—for all action conditions—significant activation in somatosensory, motor, premotor, parietal, and occipitotemporal cortex even after controlling for eye movements, we would conclude that activation differences between action and observation are not the result of eye movements but is rather the result of (actively performed) visually-guided finger movements.

### General Task Activations

Controlling for movement amplitude and eye movement parameters, the visually guided finger movements significantly activated somatosensory, motor, premotor, parietal, and occipitotemporal cortex in the four action conditions (Figure 2). In contrast, the observation of the various moving visual stimuli (hands, PL, and CU) on the screen only activated the OTC, but none of the reported MNS regions in the premotor and parietal cortex (Molenberghs et al., 2012a).

Several studies have questioned the involvement of the premotor-parietal cortical network in action observation because fMRI and PET did not all show activation in this network as clearly as in monkey experiments. There is evidence that many factors can shape and increase activity in the MNS network, such as novelty and experience (Liew et al., 2013), subtle differences in movement kinematics (Koul et al., 2018), intention and the context of observed motor action (Molenberghs et al., 2012a). In an older study, individuals watched video clips showing object manipulation by the right or left hand (Shmuelof and Zohary, 2006). The occipital cortex and caudal part of the parietal cortex demonstrated fMRI signal responses specific to the visual-field location of the clips. However, the response in anterior IPS was related to the identity of the observed hand. These “hand-specific” parietal areas also demonstrated contralateral hand specificity during self-action (i.e., object manipulation) without visual feedback. The authors concluded that the anterior IPS is involved in the observation of specific hand actions, including grasping.

Most important are the publications reporting meta-analysis of many fMRI studies in the context of action observation and execution (Molenberghs et al., 2012a; Hardwick et al., 2018). Morin and Grèzes (2008) reported that only observing biological actions with a physical target (compared to visual stimuli displaying no action at all) leads to consistent activation of the PMv (Morin and Grèzes, 2008). Caspers et al.’s (2010) meta-analysis concluded that the activation in the PMv was only for observation objects-related actions (Caspers et al., 2010).To the same conclusion came the meta-analyses on imagery, observation, and execution by Hardwick et al. (2018), who found that one of the potential factors influencing activation in the network is the involvement of an object in the observed actions (Hardwick et al., 2018). The lack of activation in the premotor cortex in our study was surprising but could be explained by the conservative statistical threshold (*p* < 0.05, FWE) applied or is due to the missing object-related action in our experiment, in which we focused on simple reaching.

We could not find any significant difference in brain activation between realistic (VH) and less realistic (SH) hand feedback, neither during action nor during observation (Figure 3). This is consistent with a previous study (Perani et al., 2001). Yet, our results are not in line with another result of the same study. According to their experiment, the observation of RH feedback yielded stronger activation compared to a coarse VH (close to a robotic hand). However, this surprising result might be explained by the fact that the presented hand belonged to a human agent (located in the scanner room) rather than the display of a VH on the screen. In early visual regions, we did not find any activation differences between baseline and action or observation. This was probably due to the static visual stimulus (blue fixation cross) that subjects saw during rest periods. In both action and observation conditions, we found stronger activation along the visual cortex comparing the two-hand to the two point-light feedback conditions (Figure 4).

## Limitations

As the four visual feedback types differed in size, this could explain some of the observed activation differences, especially in early visual regions. However, the contrast “PL > CU” conditions, comparing differently sized feedback, only evoked activation differences in the lingual gyrus and only during observation. Hence, it is unlikely that activation differences in the examined regions are driven by differences in the physical size of the stimuli. Also, the fMRI signal amplitudes in higher visual brain regions (ROI: EBA, hMT+, LOC, and LOTC) were stronger for all action than observation conditions. This result suggests that processing in these regions is associated with action understanding and cannot only reflect changes in the physical properties of the feedback types (Kilner, 2011; Wurm et al., 2017).

Second, activation differences between hands and circles might be related to differences in behavioral parameters, as observed for movement amplitude and reaction time (Brand et al., 2016). Yet, an amplitude parametric regressor of no interest was added to the first-level fMRI design to remove possible amplitude effects from the fMRI data.

Thirdly, we recorded eye movement data and found that oculomotor parameters are known to be strongly correlated in humans with both alertness (blink frequency) and visual attention (fixation duration) did not significantly differ across feedback conditions. This limits the likelihood that participants’ attention differed in the four feedback types and would elicit stronger BOLD signal amplitude for hands compared to circles. Also, we controlled for the impact of eye movement parameters on brain activation in the statistical model for the fMRI analysis, to minimize the impact of eye-movement-related activation on task-related activation.

Differences in luminance were not checked. However, the results clearly show that there was no activation difference between the VH and SH in all regions for action as well as observation, though potential differences in luminance. We did not record muscle activity by electromyography recordings. Yet, based on the sensor glove data we had detailed information on the movement trajectory and thus information about e.g., the movement extent error.

## Conclusion

Our study suggests that brain activation during visually guided finger movements depends on the visual representation of the movement on the screen. During action and observation, full-sized finger representations—whether realistic or shadow—lead to more activation in specific visual brain regions compared to point-light or cursor feedback. Our results can be important for the design of future computer-interactive and/or virtual-reality augmented training and rehabilitation systems.

## Data Availability Statement

The datasets generated for this study are available on request to the corresponding author.

## Ethics Statement

The studies involving human participants were reviewed and approved by Kantonale Ethikkommission Zürich, Zürich. The patients/participants provided their written informed consent to participate in this study.

## Author Contributions

The study was conceived by KE and M-CH-R. The data were collected by JB, MP, and LM. The statistical analyses were conducted by JB and LM. The figures were created by JB and LM. The article was written by JB and LM with input from M-CH-R, MP, and KE. All authors contributed to the article and approved the submitted version.

## Conflict of Interest

The authors declare that the research was conducted in the absence of any commercial or financial relationships that could be construed as a potential conflict of interest.

The handling Editor declared a shared affiliation, though no other collaboration, with the authors at time of review.

## Footnotes

^1^http://www.5dt.com

^2^http://www.unity3d.com

^3^http://www.sr-research.com

^4^www.fil.ion.ucl.ac.uk/spm

^5^http://marsbar.sourceforge.net
